# Population structure and molecular genetic characterization of clinical *Candida tropicalis* isolates from a tertiary-care hospital in Kuwait reveal infections with unique strains

**DOI:** 10.1371/journal.pone.0182292

**Published:** 2017-08-30

**Authors:** Khaled Al-Obaid, Mohammad Asadzadeh, Suhail Ahmad, Ziauddin Khan

**Affiliations:** 1 Microbiology, Department of Medical Laboratories, Al-Amiri Hospital, Sharq, Kuwait; 2 Department of Microbiology, Faculty of Medicine, Kuwait University, Safat, Kuwait; Wadsworth Center, UNITED STATES

## Abstract

*Candida tropicalis* is a frequently isolated yeast species causing bloodstream, urinary tract and other infections particularly in patients admitted to intensive care units (ICUs) and those requiring prolonged urinary catheterization (UC) or receiving broad-spectrum antibiotics (BSA). This study investigated clinical characteristics and genetic relatedness among *C*. *tropicalis* strains isolated from patients at Al-Amiri Hospital in Kuwait. *C*. *tropicalis* strains (n = 63) isolated from blood, genito-urinary, respiratory (RT) and digestive (GIT) tracts and wound sites from 54 patients were used. All isolates were phenotypically identified and tested against six antifungal drugs by using Vitek 2 system. Molecular identification was performed by PCR amplification of rDNA. Fingerprinting was achieved by 6-loci-based multilocus sequence typing (MLST) and data were analyzed by BioNumerics software for phylogenetic relationships. Patients mean age was >65 years and >20% patients were hospitalized in ICUs. Most patients had underlying conditions that included UC, BSA, diabetes and RT/GIT abnormalities. Most candiduria cases had UC, ureteric stent or suprapubic catheters. All isolates were identified as *C*. *tropicalis* by Vitek 2 and by species-specific PCR. Sixty-two isolates were susceptible to all tested antifungal drugs. MLST identified 59 diploid sequence types (DSTs) including 54 newly-identified DSTs. *C*. *tropicalis* isolates from multiple sites of same patient usually belonged to different DSTs. Interestingly, 56 of 57 isolates from 48 patients belonged to unique genotypes. Only six isolates from six patients belonged to three DSTs (clusters), however, *C*. *tropicalis* strains in each cluster were isolated >3 months apart. Our data show diverse origins of *C*. *tropicalis* infections in Kuwait as most isolates were unique strains. There was no obvious correlation between cluster isolates with time of isolation and/or hospital ward of their origin. This study presents the first MLST analysis of *C*. *tropicalis* isolates from Middle East and may be useful for studying genetic relationships among global *C*. *tropicalis* strains.

## Introduction

*Candida* spp. are important constituent of human microbial flora of skin, mucous membranes of mouth and vagina and gastrointestinal tract. They are also opportunistic human pathogens. The infections range in severity from mild superficial infections to life-threatening bloodstream and invasive infections inflicting considerable morbidity and mortality, particularly in immunocompromised and hospitalized patients [1, 2]. *Candida* spp. are now the fourth most common cause of all bloodstream infections and the third most common cause of bloodstream infections in patients in the intensive care unit (ICU) in many tertiary-care hospitals across the world with an attributable mortality of 15–35% in adults and 10–15% in neonates [3–5]. Nearly 90% of invasive *Candida* infections are caused by only four species or species complexes which include *Candida albicans*, *Candida glabrata*, *Candida parapsilosis*, and *Candida tropicalis* [3–6]. *Candida tropicalis* is the second most frequently isolated *Candida* from bloodstream in many countries and is also the leading cause of nosocomial fungemia and invasive fungal infections in patients with hematologic and other malignancies in several countries [5–10]. *C*. *tropicalis* infections are more common in older patients admitted to ICUs and those requiring prolonged urinary catheterization (UC) or receiving broad-spectrum antibiotics (BSA) [7, 11]. Antifungal drug susceptibility testing of *Candida* spp. isolates have also shown that the frequency of fluconazole-resistant (including isolates that show intermediate susceptibility) *C*. *tropicalis* strains is high (~10%) and is comparable to that of *C*. *glabrata* isolates in some Asian countries [6, 12, 13].

Candiduria, *Candida* colonization or infection of the urinary tract, is also common among hospitalized patients with predisposing diseases such as diabetes mellitus and abnormalities of the kidney and urinary tract (urinary tract obstruction, surgery or instrumentation), especially those in the ICU as they acquire several common risk factors such as urinary drainage devices, parenteral nutrition, mechanical ventilation and broad-spectrum antibacterial therapy [11, 14]. *Candida* spp. are third most common organisms isolated from urine samples of hospitalized patients and age older than 65 years, female sex, diabetes mellitus and urinary tract abnormalities are independent risk factors for developing candiduria [11, 14–16]. In many tertiary care facilities, nearly 10% of all cultures from urine samples yield *Candida* spp. isolates with *C*. *tropicalis* as the second or third most common *Candida* species causing candiduria [11, 15–19]. Treatment of candiduria in patients with symptoms of urinary tract infection (UTI) is beneficial and mortality rates are higher in hospitalized patients who have candiduria compared to similar patients without candiduria [7, 11, 14, 20].

The origin of nosocomial *Candida* infections could be endogenous strains brought into the hospital environment by the patients themselves or exogenous strains transmitted to the patients from biomedical devices, contaminated infusates, hospital surroundings and health care workers [21, 22]. Two molecular typing techniques, capable of discriminating closely related but non-identical isolates, multilocus sequence typing (MLST) based on single-nucleotide polymorphisms within housekeeping genes with discriminatory power of 0.99 and multilocus microsatellite typing (MLMT) based on microsatellite length polymorphism with discriminatory power of 0.97 have mostly been used in recent years for determining genetic relatedness among clinical *C*. *tropicalis* isolates [23–25]. Of these, MLST is a standardized scheme based on six conserved housekeeping genes and has a publicly accessible and curated online *C*. *tropicalis* MLST database (http://pubmlst.org/ctropicalis/) for worldwide comparisons. Previous studies based on MLMT and MLST have shown that most *C*. *tropicalis* infections develop as a consequence of endogenous colonization with this species, however, outbreaks of *C*. *tropicalis* candiduria due to poor management of medical waste (urine) in ICUs have also been described [11, 26–29]. However, such studies have not been carried out from any of the Middle Eastern countries.

This study performed molecular genetic characterization of *C*. *tropicalis* strains isolated at a major tertiary-care hospital in Kuwait by MLST to ascertain nosocomial clusters/common source of infection among patients and compared MLST data with data obtained previously from a global collection of isolates to better understand the population structure of *C*. *tropicalis*.

## Materials and methods

### Reference strains and clinical isolates of *C*. *tropicalis*

Reference strains of *C*. *tropicalis* (ATCC 750), *Candida viswanathii* (CBS 1924), *C*. *albicans* (ATCC 90028), *Candida dubliniensis* (CD36), *C*. *parapsilosis* (ATCC 22019), *Candida orthopsilosis* (ATCC 96139), *Candida metapsilosis* (ATCC 96143), *Lodderomyces elongisporus* (CBS 2605), *C*. *glabrata* (ATCC 15545), *Candida nivariensis* (CBS 9983), *Candida bracarensis* (CBS 10154), *Candida krusei* (ATCC 6258) and *Candida haemulonii* (CBS 5149) were used. Fifty-seven *C*. *tropicalis* isolates obtained from 48 consecutive patients at Al-Amiri, a major tertiary-care hospital in Kuwait, during an 8-month period (March 2015 to October, 2015) were used. Additionally, six isolates collected eight months later from six patients were also used for comparison purposes. Most (54 of 63, 86%) isolates originated from genito-urinary and respiratory (RT)/gastro-intestinal (GIT) tract specimens including urine (n = 38), vaginal swab (n = 1), sputum (n = 1), endotracheal aspirate (n = 2), oral/throat swab (n = 4), tracheostomy (n = 1), percutaneous endoscopic gastrostomy (n = 4), stool/anal swab (n = 2) and abdominal fluid (n = 1). The remaining nine isolates were obtained from bloodstream (n = 5) and skin wound swabs (n = 4). Thus, a total of 63 *C*. *tropicalis* isolates from 54 patients were used. The source, date of isolation and underlying conditions among patients yielding *C*. *tropicalis* isolates are provided in S1 Table. The clinical specimens were collected after obtaining verbal consent from patients as part of routine patient care for the isolation of fungal pathogens and the data were analyzed anonymously. The consent procedure and the study were approved by the Ethics Committee of the Faculty of Medicine, Health Sciences Center, Kuwait University (Approval no. VDR/EC/2336 dated 2-6-2015). The phenotypic identity of the isolates was initially based on assimilation profiles on commercial Vitek 2 yeast identification system (bioMérieux, Marcy-l'Étoile, France) which was used according to manufacturer’s instructions. The isolates were sub-cultured on Sabouraud dextrose agar medium for molecular identification and MLST analyses.

### DNA extraction and species-specific identification of *C*. *tropicalis* isolates

Genomic DNA was extracted from 1 ml of cell suspension in Sabouraud dextrose broth by using Gentra Puregene Yeast DNA extraction kit (Qiagen, Hilden, Germany) according to kit instructions or by the rapid method using Chelex-100 as described previously [30]. Molecular identification was performed by PCR amplification of internally transcribed spacer (ITS) region of ribosomal DNA (rDNA) by designing and using two *C*. *tropicalis*-specific primers; CTROPF (5’-TTTATTTACAGTCAAACTTGAT-3’) and CTROPR (5’-TTAAATTCTTTCAAACAAACC-3’). PCR amplification and detection of amplicons by agarose gel electrophoresis was carried out as described previously [31, 32] except that CTROPF and CTROPR primers were used. DNA sequencing of the entire ITS region (including ITS-1-5.8S rRNA-ITS-2) of rDNA was also performed, as described previously [33, 34], for five selected isolates to confirm the results of species-specific PCR amplification. Basic local alignment search tool (BLAST) (http://blast.ncbi.nlm.nih.gov/Blast.cgi?CMD=Web&PAGE_TYPE=BlastHome) searches were carried out and >99% sequence identity with reference strain of *C*. *tropicalis* (ATCC 750) were used for species identification. The ITS region sequence data have been submitted to EMBL/GenBank databases under accession numbers LT837794 to LT837798.

### Antifungal drug susceptibility testing

The susceptibility of *C*. *tropicalis* isolates against six antifungal drugs; amphotericin B, 5-flucytosine, fluconazole, voriconazole, caspofungin and micafungin was determined by using Vitek 2 AST system according to the manufacturer’s instructions (bioMérieux). The results were interpreted according to the revised interpretive susceptibility breakpoints as recommended by Clinical Laboratory Standards Institute (CLSI) [35]. Quality control was ensured by testing *C*. *albicans* ATCC 90028, *C*. *parapsilosis* ATCC 22019 and *C*. *tropicalis* ATCC 750, as recommended by CLSI.

### Fingerprinting of *C*. *tropicalis* isolates by MLST

All 63 *C*. *tropicalis* isolates were analyzed by using the MLST scheme based on PCR amplification and DNA sequencing of six housekeeping gene (*ICL1*, *MDR1*, *SAPT2*, *SAPT4*, *XYR1* and *ZWF1a*) fragments as described by Tavanti et al. [23]. However, we used new sets of primers for PCR amplification of each gene target and internal primers (to avoid any interference by primer-dimer artifacts) for more efficient sequencing of PCR amplicons [36]. The sequences and other features of the primers used for MLST are listed in S2 Table.

The PCR amplification and cycling conditions for the six gene fragments were same as described previously [31] except that gene-specific amplification primers (listed in S2 Table) were used for each gene. The amplicons were purified and subjected to bi-directional sequencing as described previously [33, 34] except that gene-specific primers (listed in S2 Table) were used as sequencing primers. Each sequence chromatogram was reviewed for heterozygous (two equally strong and overlayed fluorescence peaks) nucleotide positions. The allelic profile (allele number) for each gene and allele combinations (diploid sequence type, DST) for the six loci for each isolate were assigned or new allele and new DST numbers were provided by the curator, Prof. Frank C. Odds of the *C*. *tropicalis* MLST database (http://pubmlst.org/ctropicalis/). The phylogenetic relationship of *C*. *tropicalis* isolates was also determined. Based on the allele number for the six loci for each isolate, a dendrogram was constructed with the clustering method, using unweighted pair group method with arithmetic averages (UPGMA) settings of BioNumerics v7.5 software (Applied Maths, Saint-Martens-Latem, Belgium). The isolates belonged to the same DST when they contained the same alleles for all six loci. A cluster was defined as a group of two or more patients infected with a *C*. *tropicalis* strain belonging to the same DST. The MLST data were further analyzed for clonal clusters by using the eBURST package. The genetic relationship between the 63 isolates from Kuwait with 804 isolates in the central data library of *C*. *tropicalis* MLST database as of January 2017 was also studied by using the minimum spanning tree of the BioNumerics software. The minimum spanning tree predicts putative relationships among the isolates and records the isolates as more closely related when five of six loci are identical.

## Results

### Characteristics of patients yielding *C*. *tropicalis* isolates

We performed molecular genetic characterization and fingerprinting of 63 *C*. *tropicalis* isolates obtained from 54 patients. One isolate each was tested from 47 patients, two isolates each were obtained from five patients and three isolates each were tested from two patients. Multiple isolates from the same patient were from different anatomic sites. The majority (38 of 63, 60%) of the isolates were obtained from urine samples from patients with candiduria while only five isolates were obtained from bloodstream of candidemia patients.

The demographic details of the 54 patients yielding 63 *C*. *tropicalis* isolates were as follows. Most (48, 89%) patients were Kuwaiti nationals while six patients were non-Kuwaiti expatriate residents. The country of origin of non-Kuwaiti patients included Egypt (n = 2), Iran (n = 2), Bahrain (n = 1) and Pakistan (n = 1). The age range of the patients varied from 11 years to 97 years old. Nearly two-thirds (35 of 54, 65%) of the patients were >65 years of age and the ratio of male to female was nearly equal (28 males and 26 females). Four *C*. *tropicalis* isolates were obtained from four patients who were attending the clinic or out-patient department while the remaining 59 isolates were cultured from 50 hospitalized patients including 12 (22%) patients from an ICU, six patients from the emergency room, three patients from the dialysis unit and 29 patients from different wards in the hospital. Various underlying conditions for *Candida* infections were present in 49 of 54 patients (no information was available for four patients who attended the clinic or out-patient department and one patient who was admitted to the emergency room) and are summarized in Table 1. The most common underlying conditions were the presence of a urinary catheter, kidney disease and/or urinary tract infection in 29 patients, treatment with broad-spectrum antibiotics in 29 patients and diabetes mellitus in 24 patients. Other factors included prostate hyperplasia and/or cancer in 12 patients and gastro-intestinal bleeding, liver disease and/or abdominal surgery in 10 patients. The most common underlying condition among patients with candiduria included the presence of a urinary catheter, urinary obstruction (e.g. benign prostatic hyperplasia) and/or kidney disease and older (>65 years) age (Table 1).

**Table 1:** Common underlying conditions among patients yielding *C*. *tropicalis* isolates.

Common underlying	No. of	No. of patients
condition(s)	patients	>65 years old
Urinary catheter/urinary tract infection/kidney disease	29	20
On broad-spectrum antibiotics	29	19
Diabetes mellitus	24	18
Cancer/prostate hyperplasia	12	9
Abdominal surgery/gastro-intestinal bleeding/liver disease	11	5
Candidemia/septic shock	7	4
Chest infection/respiratory failure	7	5
Heart disease or brain injury	3	2

### Phenotypic and molecular identification of *C*. *tropicalis* isolates

Each isolate was subjected to identification by using Vitek 2 yeast identification system which identified all 63 isolates as *C*. *tropicalis*. For genotypic identification, the genomic DNA prepared from each isolate was subjected to PCR amplification of rDNA by using species-specific CTROPF and CTROPR primers. The species-specificity of the primers for *C*. *tropicalis* DNA was indicated by BLAST searches and was confirmed by lack of PCR amplification of rDNA with genomic DNA from common *Candida* species other than *C*. *tropicalis*, as expected. Only genomic DNA from reference *C*. *tropicalis* strain (ATCC 750) yielded an expected amplicon of 259 bp (S1 Fig). The genomic DNA from all 63 isolates yielded an amplicon of ~259 bp confirming the identification of all isolates as *C*. *tropicalis*. The DNA sequencing of the entire ITS region (including ITS-1-5.8S rRNA-ITS-2) of five selected isolates was also performed and showed variation at only one or two nucleotide positions (<1% difference) with the corresponding sequence from reference *C*. *tropicalis* strain (ATCC 750) further confirming the identification of all five isolates as *C*. *tropicalis*. The sequencing data also identified three ITS haplotypes among five *C*. *tropicalis* isolates (S2 Fig).

### Phenotypic antifungal drug susceptibility testing of *C*. *tropicalis* isolates

The antifungal drug susceptibility testing (AST) of all 63 *C*. *tropicalis* isolates was performed against six (amphotericin B, 5-flucytosine, fluconazole, voriconazole, caspofungin and micafungin) antifungal drugs by using the Vitek 2 system and the results of AST are presented in S3 Table. All isolates were uniformly susceptible to amphotericin B, fluconazole, voriconazole, caspofungin and micafungin. The minimum inhibitory concentration (MIC) range for amphotericin B varied between 0.25 mg/L to 0.5 mg/L only. The MIC values for fluconazole, voriconazole, caspofungin and micafungin showed no variation among the isolates (S3 Table). Only one isolate (Kw45-15) with MIC value of 16 mg/L was resistant to 5-flucytosine while the remaining 62 isolates were susceptible to 5-flucytosine.

### Genetic diversity of *C*. *tropicalis* isolates in Kuwait by MLST

The genetic diversity and population structure of *C*. *tropicalis* isolates in Kuwait was studied by MLST. All six gene fragments were successfully amplified, sequenced by using the corresponding gene-specific forward and reverse primers (S2 Table) and the DNA sequences were used to obtain the allelic profiles for each gene fragment and allele combinations to obtain DSTs from the *C*. *tropicalis* MLST database (http://pubmlst.org/ctropicalis/). A total of 88 cases of heterozygosity were detected in the six sequenced fragments and 144 polymorphic sites were identified, including 26 in *ICL1*, 36 in *MDR1*, 40 in *SAPT2*, 19 in *SAPT4*, 12 in *XYR1* and 11 in *ZWF1a*. The highest typing efficiency (1.83 genotypes per polymorphism) was obtained with *XYR1* while the lowest typing efficiency (0.27 genotypes per polymorphism) was obtained with *SAPT2*. Twenty-two new alleles were identified among *C*. *tropicalis* isolates from Kuwait during searches of the *C*. *tropicalis* MLST database (http://pubmlst.org/ctropicalis/).

The polymorphic alleles combined to form 59 DSTs among 63 *C*. *tropicalis* isolates from 54 patients. Of the 59 DSTs, only 5 DSTs (DST94, DST99, DST114, DST168 and DST238) for 6 isolates (DST238 was shared among two isolates) were present in the *C*. *tropicalis* MLST database while 54 DSTs (DST677 to DST731) for 57 isolates (DST678, DST681 and DST688 were shared among two isolates each) were new and were added to the *C*. *tropicalis* MLST database. Of the 54 new DSTs, 34 DSTs were generated by rearrangement of previously known alleles while the remaining 20 DSTs were formed due to the presence of a new allele for 1–3 genes. The allelic profile and the final DST for each isolate are shown in S1 Table.

The fingerprinting data further showed that multiple isolates from the same patient but recovered from different anatomic sites were genotypically different as they belonged to different DSTs. The seven urine *C*. *tropicalis* isolates obtained from seven candiduria patients were genotypically different than the isolates cultured from other clinical specimens of the same patient (Table 2). Six of seven candiduria patients were >65 years old while one patient was 60 years old and six of seven were male patients. Furthermore, bloodstream isolate from one patient belonged to a different DST than the isolates from urine and endotracheal aspirate from the same patient. Only two non-urine isolates from one patient and originating from the GIT (oral swab and anal swab) belonged to the same DST (Table 2). Interestingly, the two isolates from one patient (patient no. 18) differed at all six loci, two isolates from two patients (patient no. 9 and 14) differed at five loci, two isolates from one patient (patient no. 25) differed at four loci and two isolates from another patient (patient no. 30) differed at three loci. Multiple isolates from only two patients (patient no. 3 and 38) showed little or no variations (Table 2).

**Table 2:** MLST data for multiple *C*. *tropicalis* isolates from different anatomic sites of seven patients.

Patient	Clinical	Date of	Isolate	Allelic profiles for housekeeping gene fragment for						MLST-based
no.	specimen^a^	isolation	no.	*ICL1*	*MDR1*	*SAPT2*	*SAPT4*	*XYR1*	*ZWF1a*	DST^b^
3	Urine	27-03-15	Kw5-15	1	42	4	23	16	1	**690**
3	Oral swab	27-03-15	Kw6-15	1	42	1	23	16	1	**688**
3	Anal swab	27-03-15	Kw7-15	1	42	1	23	16	1	**688**
9	Urine	16-04-15	Kw13-15	1	22	12	78	60	22	**705**
9	Tracheostomy	20-04-15	Kw14-15	1	7	4	6	52	4	238
14	Urine	21-05-15	Kw19-15	1	3	3	17	57	3	168
14	Oral swab	21-05-15	Kw32-15	1	7	4	6	11	4	**684**
18	Urine	12-06-15	Kw23-15	3	145	3	17	6	1	**725**
18	Throat swab	12-06-15	Kw24-15	1	96	34	7	92	34	**693**
25	Urine	11-07-15	Kw34-15	1	7	4	6	11	4	**681**
25	Stool	11-07-15	Kw35-15	1	139	4	17	6	3	**711**
30	Urine	04-08-15	Kw40-15	1	4	3	23	13	2	**680**
30	Wound swab	04-08-15	Kw41-15	1	140	3	23	132	7	**712**
38	Urine	17-09-15	Kw50-15	1	1	10	21	6	1	**702**
38	Blood	17-09-15	Kw51-15	1	1	3	23	6	1	**703**
38	ET aspirate	17-09-15	Kw52-15	1	1	10	1	6	1	**677**

^a^ET aspirate, endotracheal aspirate.

^b^MLST-based DST, multi locus sequence type-based diploid sequence type; new DSTs detected in this study are shown in bold and DSTs shared between two isolates obtained from same or different patients from Kuwait are underlined.

**Fig 1:**
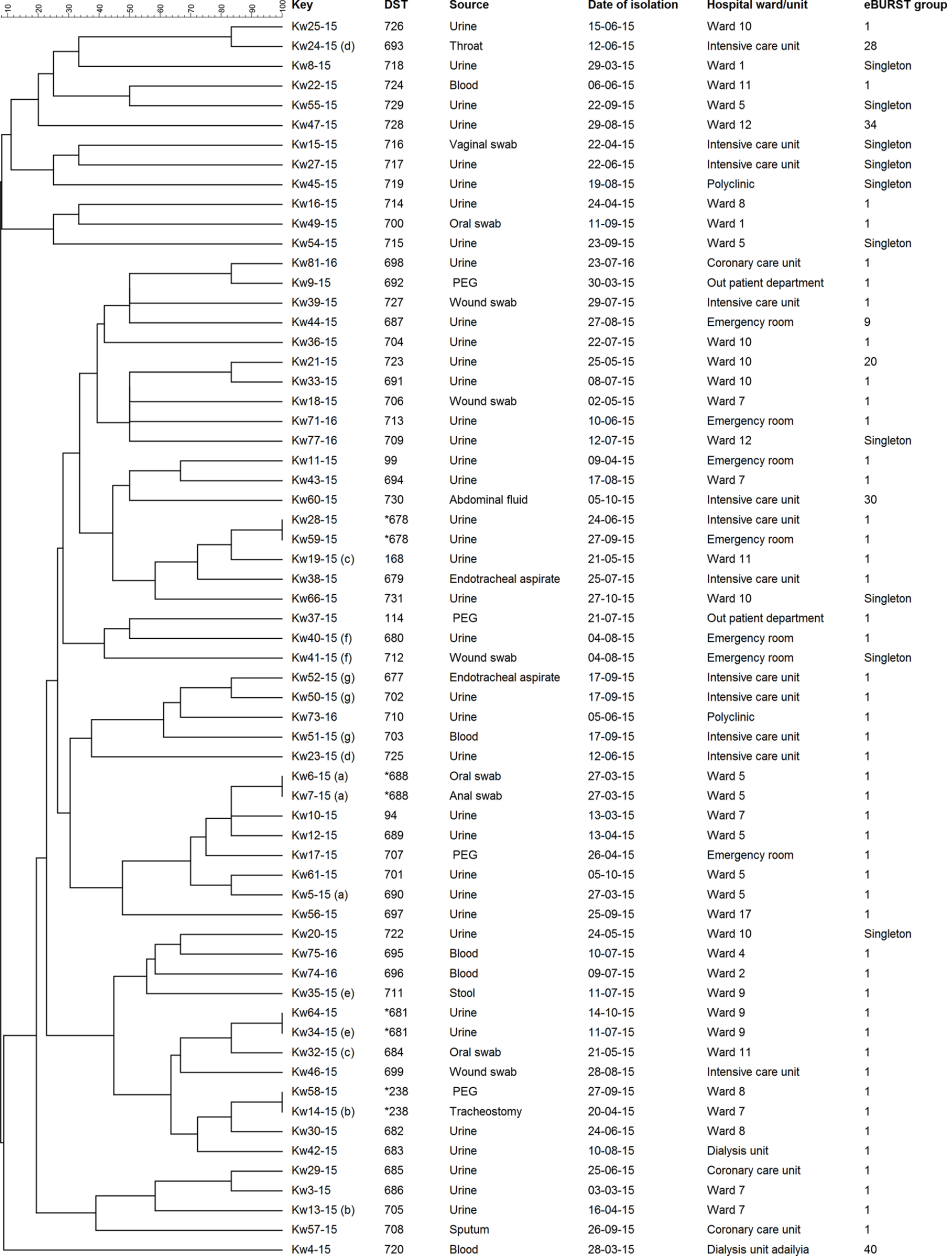
An UPGMA-derived dendrogram based on allelic profile of 6 gene fragments from 63 *C*. *tropicalis* isolate from Kuwait. Similarity is presented in percentages using the scale bar in the upper left corner. The columns from left to right include, isolate number, MLST-based diploid sequence type (DST), clinical specimen yielding the isolate, data of isolation, hospital ward/unit where the patients were housed and eBURST group. Repeat isolates from the same patient are indicated by alphabets within brackets and DSTs for cluster isolates are shown by an asterisk (*) before the DST.

The genetic association between the DSTs of *C*. *tropicalis* isolates from Kuwait was determined by construction of an unrooted phylogenetic tree based on MLST data. The dendrogram (Fig 1) showed that most (55 of 63, 87%) of the isolates were dispersed as unrelated singletons belonging to a single unique DST while only four DSTs were shared, each among two isolates. Majority of the isolates differed at two or more loci. No obvious relationship was found between DSTs and specimen types or patient’s nationality. Among the four cluster DSTs (DST238, DST678, DST681 and DST688), DST688 actually involved two isolates from related clinical specimens from the same patient. The remaining three clusters included *C*. *tropicalis* isolates obtained from similar clinical specimens from two unrelated patients in each case. The clinical and epidemiological data from the patients yielding cluster isolates were compared to ascertain if the cluster isolates represented cross-transmission of infection and are presented in Table 3. The data showed that cluster isolates in each case were isolated at different time points which varied from >3 months to >5 months. Furthermore, the isolates were obtained from patients that were hospitalized in different wards in two of the three clusters.

**Table 3:** Clinical and epidemiological characteristics of patients yielding *C*. *tropicalis* isolates belonging to the three MLST-based clusters.

Cluster	Diploid sequence	Source of	Date of	Patient	Hospital
no.	type (DST)	isolation^a^	isolation	no.	ward
1	DST238	Tracheostomy	20-04-15	9	7
	DST238	PEG	27-09-15	43	8
2	DST678	Urine	24-06-15	21	Intensive care unit
	DST678	Urine	27-09-15	44	Emergency room
3	DST681	Urine	11-07-15	25	9
	DST681	Urine	14-10-15	47	9

^a^PEG, percutaneous endoscopic gastrostomy.

**Fig 2:**
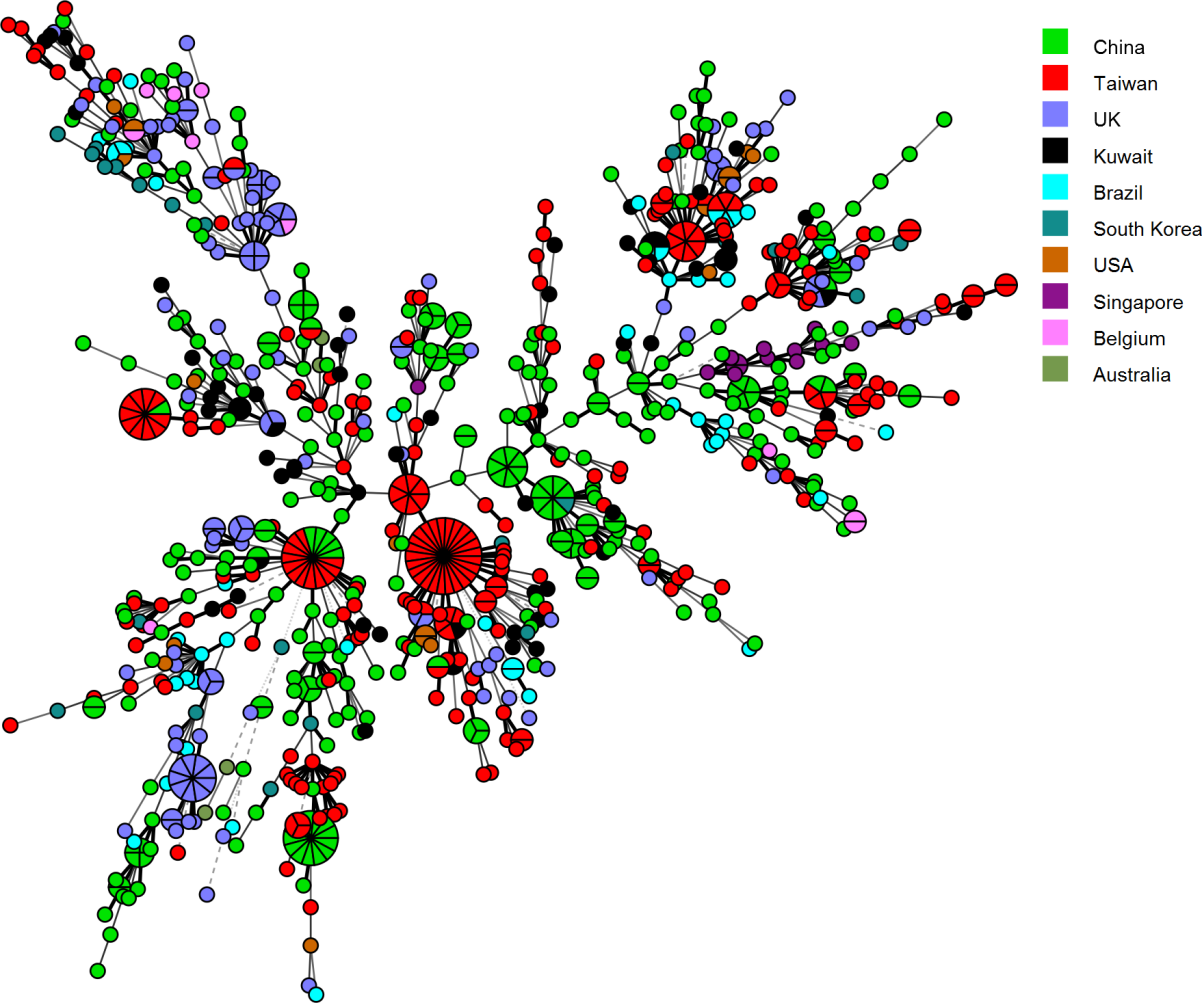
Minimum spanning tree showing relationship of 63 *C*. *tropicalis* isolates from Kuwait with 804 isolates from other countries available from the MLST website as of January 2017. Each circle corresponds to a unique genotype, and lines between circles represent relative distance between isolates. The sizes of the circles correspond to the number of isolates of the same genotype (DST). Connecting lines correspond to the number of allele differences between genotypes, with a solid thick line connecting genotypes that differ in one locus, a solid thin line connecting genotypes that differ in two-three loci, a dashed line connecting genotypes that differ in four loci, and a dotted line connecting genotypes that differ in more than four loci.

The population structure of *C*. *tropicalis* isolates from Kuwait was further studied by determining genetic relationship between the 63 isolates from Kuwait with 804 isolates (as of January 2017) in the central data library of *C*. *tropicalis* MLST database by using the eBURST and minimal spanning tree algorithm of the BioNumerics software. The minimal spanning tree is depicted in Fig 2. Of the 59 DSTs detected among 63 isolates, 49 DSTs were grouped into seven groups (groups 1, 9, 20, 28, 30, 34 and 40), and 10 DSTs were identified as singletons. Interestingly, 43 of 49 (88%) clonal group isolates belonged to eBURST group 1 that also included most of the isolates from other geographical locations in the data base. Furthermore, most of the isolates from Kuwait clustered with isolates from several Asian (China, Taiwan and South Korea) countries while few isolates also clustered with *C*. *tropicalis* isolates from the United Kingdom and Brazil (Fig 2).

## Discussion

This study is the first MLST analysis to characterize clinical *C*. *tropicalis* isolates from a Middle Eastern country and compared the patterns of variation with those in the MLST database representing isolates from other geographic regions. A total of 63 isolates obtained from 54 patients were analyzed. Consistent with earlier reports, most candiduria patients had several underlying conditions for *Candida* infections including age older than 65 years, urinary drainage devices, urinary obstruction (e.g. prostate hyperplasia) and/or kidney disease, prior use of broad-spectrum antibiotics and diabetes mellitus, however, unlike other studies, male patients outnumbered females [14–18]. Furthermore, all candidemia cases were associated with intravascular catheters.

All 63 isolates were phenotypically identified by Vitek 2 yeast identification system and genotypically by PCR amplification of rDNA by using novel *C*. *tropicalis*-specific (CTROPF and CTROPR) primers as *C*. *tropicalis*. The ITS sequencing data from five selected isolates identified three haplotypes showing genotypic heterogeneity among *C*. *tropicalis* isolates. Three ITS haplotypes were also identified among 48 *C*. *tropicalis* isolates while four haplotypes were identified for D1/D2 domains of rDNA in a recent study from Beijing, China [25]. The ITS region sequences show greater variation than D1/D2 domain sequences within rDNA [37, 38]. It is, therefore, probable that sequencing of a larger number of *C*. *tropicalis* isolates from Kuwait and other geographical locations may identify additional ITS haplotypes as it was recently observed for clinical *C*. *dubliniensis* isolates [39–41].

The AST data obtained by using the Vitek 2 AST system which is considered comparable to the reference broth microdilution method [42] showed that all 63 *C*. *tropicalis* isolates were uniformly susceptible to amphotericin B, fluconazole, voriconazole, caspofungin and micafungin with MIC values showing little variation among the isolates. Thus, antifungal drug resistance was not a serious concern among *C*. *tropicalis* isolates at Al-Amiri Hospital in Kuwait. This could be due to relatively low antifungal consumption for prophylactic or empiric use as there are no hematology or oncology wards in our hospital. The findings are similar to two other studies from China and Taiwan which showed that resistance to fluconazole and other antifungal agents was rare in *C*. *tropicalis* isolates [26, 43]. However, they are contrary to several other reports documenting the presence of resistance to azoles and/or other antifungal agents among >20% of clinical *C*. *tropicalis* isolates from some geographical locations including some locations in China [44–50]. Resistance to antifungal agents in *Candida* spp. develops during therapy as a result of selective drug pressure [5, 6, 12, 42, 45, 48], however, one recent report has suggested that applications of triazole fungicides in indoor/outdoor environment may have selected resistant strains of *C*. *tropicalis* which were passed onto human hosts in Hainan, China, a scenario similar to the opportunistic human fungal pathogen *Aspergillus fumigatus* [49]. Our data, however, do not support this scenario as triazole-resistant *A*. *fumigatus* have previously been isolated readily from both, environmental and clinical specimens [51–53] but azole resistance in *Candida* spp. has rarely been detected in Kuwait [30, 41, 54–56]. The data further showed that only one isolate (Kw45-15) was resistant to 5-flucytosine even though this drug is rarely used in Kuwait. Similar to our results, all *C*. *tropicalis* isolates from candidemia patients in the Spanish study were also susceptible to fluconazole and other antifungals while one isolate was resistant to 5-flucytosine [7]. On the contrary, in a survey of fungemia cases due to *C*. *tropicalis* conducted in Paris, France, and among serial *C*. *tropicalis* isolates from ICU patients in Taipei, Taiwan, ~35% of the isolates were resistant to 5-flucytosine [43, 57].

The MLST identified 144 polymorphic sites with maximum number (n = 40) of sites obtained for *SAPT2* among of 63 *C*. *tropicalis* isolates from Kuwait. Furthermore, the maximum typing efficiency (number of genotypes/polymorphic site) was obtained with *XYR1* while the lowest typing efficiency was obtained with *SAPT2*. Previous studies from other geographical locations have reported variations in the number of polymorphic sites with different loci exhibiting maximum number of polymorphic sites as well as variations in typing efficiencies [23, 26, 45–47, 49]. These findings suggest genetic variations in *C*. *tropicalis* strains at different geographical locations. The MLST data further showed that *C*. *tropicalis* isolates from Kuwait contained abundant worldwide alleles for most loci but also exhibited novel genetic variations as 22 new alleles were also identified [http://pubmlst.org/ctropicalis/].

*C*. *tropicalis* isolates in Kuwait exhibited high genetic diversity as MLST identified 59 DSTs among 63 isolates including 54 new DSTs. All five (DST94, DST99, DST114, DST168 and DST238) previously described DSTs are rare and include only 2–5 isolates from China, Taiwan, UK, Colombia and Brazil. Although DST168, previously reported only from Taiwan, was found in Kuwait, DST164 which was also detected in Taiwan and was previously shown to be associated with 5-flucytosine resistance [43] was not detected in our study and the single 5-flucytosine-resistant isolate (Kw45-15) from Kuwait belonged to a new DST (DST719). Furthermore, DST238, which was shared between two isolates originating from vaginal and percutaneous endoscopic gastrostomy specimens in Kuwait, has previously been described for two bloodstream isolates from Brazil [46]. Our data reinforce previous observations suggesting lack of association between different DSTs with antifungal resistance, specimen type or geographical location and support both shared and unique features among geographic populations of *C*. *tropicalis* [23, 26, 46, 49].

Among the seven patients yielding multiple isolates from different anatomic sites at the same or nearly same time, isolates from only two patients (patient no. 3 and 38) belonged to identical or highly related (with five or all 6 identical loci) DSTs while multiple isolates from the remaining five patients belonged to unrelated (with 3 or 4 different loci) or highly unrelated (with 5 or all 6 different loci) DSTs indicating microvariation as well as macrovariation in the nucleotide polymorphisms of the six loci. Other MLST studies involving multiple isolates from individual patients have also reported that some patients maintain the same *C*. *tropicalis* clone while isolates in other patients either undergo microvariation in one or two genes yielding related DSTs or macrovariation in nearly all six genes yielding unrelated and different DSTs [23, 43, 46, 47]. The MLST data from Brazil also showed that *C*. *tropicalis* isolates from the catheter were genetically different than the bloodstream isolate(s) in eight of nine patients indicating that different clonal populations co-exist within the same host [46]. Taken together, these findings suggest that *C*. *tropicalis* isolates undergo genetic alterations in the six genes used for MLST under the application of host and other (such as antifungal) stresses. This is in contrast to *C*. *albicans* where sequential/multiple clinical isolates from various anatomical sites of the same patient have been shown by MLST analyses to be genetically identical or highly similar [56, 58–62].

The phylogenetic tree based on MLST data showed that most (55 of 63, 87%) of the isolates were unique strains while only four DSTs were shared, each among two isolates. The clinical and epidemiological data from the patients yielding cluster isolates showed that cluster isolates in each case were isolated at different time points. Furthermore, the isolates were obtained from patients hospitalized in different wards in two of the three clusters. These findings suggest that intrahospital transmission of *C*. *tropicalis* does not occur or occurs rarely among hospitalized patients in Kuwait. Intrahospital transmission of *C*. *albicans* was also not found in a recent study involving candidemia patients in Kuwait [56].

## Conclusions

The data presented in this study have shown that most *C*. *tropicalis* strains were isolated from patients whose mean age was >65 years. Common underlying conditions detected among patients yielding *C*. *tropicalis* isolates included urinary catheter, treatment with broad-spectrum antibiotics, diabetes mellitus and RT/GIT abnormalities. Most candiduria cases had urinary catheter, ureteric stent or suprapubic catheters. All isolates were susceptible to five of six antifungal drugs and only one isolate was resistant to 5-flucytosine. MLST identified 59 DSTs including 54 newly-identified DSTs. *C*. *tropicalis* isolates from multiple sites of same patient usually belonged to different DSTs. Interestingly, 56 of 57 isolates from 48 patients belonged to unique genotypes. Our data show diverse origins of *C*. *tropicalis* infections in Kuwait as most isolates were unique strains, however, microvariation and macrovariation were also noted among *C*. *tropicalis* isolates recovered from the same patients. Only six isolates from six patients belonged to three DSTs (clusters), however, *C*. *tropicalis* strains in each cluster were isolated >3 months apart. There was no obvious correlation between cluster isolates with respect to the time of isolation and/or hospital ward of their origin. The study has also provided information on MLST-based DSTs found among clinical *C*. *tropicalis* isolates for the first time from a Middle Eastern country. New DSTs have been added to the growing list in the MLST central database that will be useful for further studies on genetic relationship and global molecular epidemiology of *C*. *tropicalis* strains.

## Supporting information

S1 Table Source of isolation, clinical characteristics and MLST data for 63 *C. tropicalis* isolates obtained from 54 patients analyzed in this study. (DOCX)

S2 Table Salient features of various oligonucleotide primers used during MLST of *C. tropicalis* isolates in this study. (DOCX)

S3 Table Antifungal drug susceptibility testing data and the minimum inhibitory concentration (MIC) range, MIC50 and MIC90 values for 63 *C. tropicalis* isolates. (DOCX)

S1 Fig Agarose gel of PCR products using *C. tropicalis*-specific (CTROPF and CTROPR) primers and genomic DNA from reference strains of *C. albicans* (Lane CA), *C. dubliniensis* (Lane CD), *C. tropicalis* (Lane CT), *C. viswanathii* (Lane CV), *C. parapsilosis* (Lane CP), *C. orthopsilosis* (Lane CO), *L. elongisporus* (Lane LE), *C. glabrata* (Lane CG), *C. nivariensis* (Lane CN) and *C. bracarensis* (Lane CB). (DOCX)

S2 Fig An UPGMA-derived dendrogram with Tamura-Nei parameters based on ITS regions of rDNA sequence data from five *C. tropicalis* isolates from Kuwait together with reference *C. tropicalis* strain ATCC 750. (DOCX)
