# Supplementary material for: Population structure and molecular genetic characterization of clinical Candida tropicalis isolates from a tertiary-care hospital in Kuwait reveal infections with unique strains
Source: PLoS One. 2017 Aug 30;12(8):e0182292. doi: 10.1371/journal.pone.0182292 (PMC5576731; doi:10.1371/journal.pone.0182292)
Supplement: S1 Table — (DOCX) [file pone.0182292.s001.docx]

**S1 Table**. **Source of isolation, clinical characteristics and MLST data for 63 *C. tropicalis* isolates obtained from 54 patients analyzed in this study.**

| **Serial** | **Patient** | **Main risk factors and** | **Clinical** | **Date of** | **Isolate** | **Allelic profiles for housekeeping gene fragment for** | | | | | | **MLST-based** |
| --- | --- | --- | --- | --- | --- | --- | --- | --- | --- | --- | --- | --- |
| **no.** | **no.^a^** | **underlying conditions^b^** | **specimen^c^** | **isolation** | **no.** | ***ICL-1*** | ***MDR1*** | ***SAPT2*** | ***SAPT4*** | ***XYR1*** | ***ZWF1A*** | **DST^d^** |
| 1 | 1 | UC, UTI, KD, US | Urine | 03-03-15 | Kw3-15 | 1 | 22 | 12 | 17 | 2 | 22 | **686** |
| 2 | 2 | KD, DM, central line | Blood | 28-03-15 | Kw4-15 | 36 | 135 | 3 | 19 | 8 | 46 | **720** |
| 3 | **3** | UC, BSA, BSI, GI bleeding | Urine | 27-03-15 | Kw5-15 | 1 | 42 | 4 | 23 | 16 | 1 | **690** |
| 4 | **3** | UC, BSA, BSI, GI bleeding | Oral swab | 27-03-15 | Kw6-15 | 1 | 42 | 1 | 23 | 16 | 1 | **688** |
| 5 | **3** | UC, BSA, BSI, GI bleeding | Anal swab | 27-03-15 | Kw7-15 | 1 | 42 | 1 | 23 | 16 | 1 | **688** |
| 6 | 4 | UC, BSA, bladder cancer | Urine | 29-03-15 | Kw8-15 | 28 | 136 | 4 | 7 | 92 | 7 | **718** |
| 7 | 5 | DM, on PEG feeding | PEG | 30-03-15 | Kw9-15 | 1 | 67 | 3 | 19 | 2 | 1 | **692** |
| 8 | 6 | BSA | Urine | 13-03-15 | Kw10-15 | 1 | 42 | 1 | 23 | 9 | 1 | 94 |
| 9 | 7 | UTI | Urine | 09-04-15 | Kw11-15 | 1 | 44 | 3 | 7 | 38 | 3 | 99 |
| 10 | 8 | UC, UTI, DM, BSA, PH | Urine | 13-04-15 | Kw12-15 | 1 | 42 | 1 | 23 | 54 | 1 | **689** |
| 11 | **9** | UC, DM, chest infection | Urine | 16-04-15 | Kw13-15 | 1 | 22 | 12 | 78 | 60 | 22 | **705** |
| 12 | **9** | UC, DM, chest infection | Tracheostomy | 20-04-15 | Kw14-15 | 1 | 7 | 4 | 6 | 52 | 4 | 238 |
| 13 | 10 | Respiratory failure | Vaginal swab | 22-04-15 | Kw15-15 | 9 | 90 | 49 | 7 | 62 | 3 | **716** |
| 14 | 11 | DM, BSA, chest infection | Urine | 24-04-15 | Kw16-15 | 3 | 137 | 1 | 10 | 27 | 6 | **714** |
| 15 | 12 | Infected PEG | PEG | 26-04-15 | Kw17-15 | 1 | 42 | 1 | 74 | 16 | 1 | **707** |
| 16 | 13 | KD | Wound swab | 02-05-15 | Kw18-15 | 1 | 31 | 3 | 75 | 9 | 1 | **706** |
| 17 | **14** | UC, UTI, KD, DM, BSA, PH | Urine | 21-05-15 | Kw19-15 | 1 | 3 | 3 | 17 | 57 | 3 | 168 |
| 18 | **14** | UC, UTI, KD, DM, BSA, PH | Oral swab | 21-05-15 | Kw32-15 | 1 | 7 | 4 | 6 | 11 | 4 | **684** |
| 19 | 15 | UC, DM, BSA | Urine | 24-05-15 | Kw20-15 | 1 | 7 | 4 | 6 | 2 | 3 | **722** |
| 20 | 16 | UC, KD, BSA | Urine | 25-05-15 | Kw21-15 | 1 | 74 | 3 | 10 | 54 | 1 | **723** |
| 21 | 17 | KD, sepsis | Blood | 06-06-15 | Kw22-15 | 39 | 144 | 3 | 7 | 92 | 32 | **724** |
| 22 | **18** | BSA | Urine | 12-06-15 | Kw23-15 | 3 | 145 | 3 | 17 | 6 | 1 | **725** |
| 23 | **18** | BSA | Throat swab | 12-06-15 | Kw24-15 | 1 | 96 | 34 | 7 | 92 | 34 | **693** |
| 24 | 19 | UC, DM, BSA | Urine | 15-06-15 | Kw25-15 | 35 | 96 | 34 | 7 | 92 | 34 | **726** |
| 25 | 20 | No Information | Urine | 22-06-15 | Kw27-15 | 25 | 138 | 50 | 7 | 6 | 3 | **717** |
| 26 | 21 | UC, DM, BSA | Urine | 24-06-15 | Kw28-15 | 1 | 3 | 3 | 17 | 57 | 7 | **678** |
| 27 | 22 | UC, DM, PH, septic shock | Urine | 25-06-15 | Kw29-15 | 1 | 22 | 12 | 17 | 2 | 7 | **685** |
| 28 | 23 | UC, UTI, DM, PH, BSA | Urine | 24-06-15 | Kw30-15 | 1 | 7 | 3 | 6 | 52 | 4 | **682** |
| 29 | 24 | No Information | Urine | 08-07-15 | Kw33-15 | 1 | 50 | 3 | 10 | 54 | 1 | **691** |
| 30 | **25** | UC, DM, BSA | Urine | 11-07-15 | Kw34-15 | 1 | 7 | 4 | 6 | 11 | 4 | **681** |
| 31 | **25** | UC, DM, BSA | Stool | 11-07-15 | Kw35-15 | 1 | 139 | 4 | 17 | 6 | 3 | **711** |
| 32 | 26 | UC, DM, BSA | Urine | 22-07-15 | Kw36-15 | 1 | 7 | 46 | 10 | 2 | 1 | **704** |
| 33 | 27 | No Information | PEG | 21-07-15 | Kw37-15 | 1 | 4 | 22 | 23 | 43 | 9 | 114 |
| 34 | 28 | KD, BSA, PH, chest infection | ET aspirate | 25-07-15 | Kw38-15 | 1 | 3 | 3 | 17 | 6 | 3 | **679** |
| 35 | 29 | UC, BSA, perforated colon | Wound swab | 29-07-15 | Kw39-15 | 1 | 42 | 3 | 10 | 2 | 32 | **727** |
| 36 | **30** | DM, Cancer, colon resected | Urine | 04-08-15 | Kw40-15 | 1 | 4 | 3 | 23 | 13 | 2 | **680** |
| 37 | **30** | DM, Cancer, colon resected | Wound swab | 04-08-15 | Kw41-15 | 1 | 140 | 3 | 23 | 132 | 7 | **712** |
| 38 | 31 | UTI, KD, DM | Urine | 10-08-15 | Kw42-15 | 1 | 7 | 3 | 9 | 52 | 4 | **683** |
| 39 | 32 | SC, BSA, brain injury | Urine | 17-08-15 | Kw43-15 | 1 | 117 | 3 | 7 | 9 | 3 | **694** |
| 40 | 33 | UTI | Urine | 27-08-15 | Kw44-15 | 1 | 34 | 3 | 13 | 2 | 2 | **687** |
| 41 | 34 | Pregnancy, antenatal screening | Urine | 19-08-15 | Kw45-15 | 31 | 131 | 1 | 15 | 6 | 3 | **719** |
| 42 | 35 | Chest infection | Wound swab | 28-08-15 | Kw46-15 | 1 | 7 | 3 | 44 | 11 | 4 | **699** |
| 43 | 36 | UC, KD, DM, OC, sepsis | Urine | 29-08-15 | Kw47-15 | 40 | 146 | 51 | 7 | 9 | 1 | **728** |
| 44 | 37 | UC, BSA, MV, sepsis | Oral swab | 11-09-15 | Kw49-15 | 3 | 7 | 1 | 7 | 35 | 4 | **700** |
| 45 | **38** | Candidemia, septic shock | Urine | 17-09-15 | Kw50-15 | 1 | 1 | 10 | 21 | 6 | 1 | **702** |
| 46 | **38** | Candidemia, septic shock | Blood | 17-09-15 | Kw51-15 | 1 | 1 | 3 | 23 | 6 | 1 | **703** |
| 47 | **38** | Candidemia, septic shock | ET aspirate | 17-09-15 | Kw52-15 | 1 | 1 | 10 | 1 | 6 | 1 | **677** |
| 48 | 39 | BSA, Liver cancer | Urine | 23-09-15 | Kw54-15 | 5 | 141 | 1 | 10 | 2 | 32 | **715** |
| 49 | 40 | UC, DM, BSA, PH | Urine | 22-09-15 | Kw55-15 | 41 | 66 | 3 | 7 | 9 | 32 | **729** |
| 50 | 41 | BSA, Liver transplant | Urine | 25-09-15 | Kw56-15 | 1 | 42 | 1 | 14 | 54 | 3 | **697** |
| 51 | 42 | DM, BSA | Sputum | 26-09-15 | Kw57-15 | 1 | 46 | 12 | 76 | 60 | 9 | **708** |
| 52 | 43 | Heart disease | PEG | 27-09-15 | Kw58-15 | 1 | 7 | 4 | 6 | 52 | 4 | 238 |
| 53 | 44 | UTI, chest infection | Urine | 27-09-15 | Kw59-15 | 1 | 3 | 3 | 17 | 57 | 7 | **678** |
| 54 | 45 | BSA, colon cancer, AD | ABD fluid | 05-10-15 | Kw60-15 | 1 | 9 | 3 | 6 | 85 | 3 | **730** |
| 55 | 46 | DM, BSA, Prostate cancer | Urine | 05-10-15 | Kw61-15 | 1 | 42 | 4 | 23 | 9 | 1 | **701** |
| 56 | 47 | DM, heart disease | Urine | 14-10-15 | Kw64-15 | 1 | 7 | 1 | 6 | 11 | 4 | **681** |
| 57 | 48 | UC, BSA, Bronchial asthma | Urine | 27-10-15 | Kw66-15 | 1 | 69 | 3 | 17 | 9 | 3 | **731** |
| 58 | 49 | No information | Urine | 10-06-16 | Kw71-16 | 1 | 141 | 3 | 8 | 3 | 1 | **713** |
| 59 | 50 | No information | Urine | 05-06-16 | Kw73-16 | 1 | 116 | 10 | 77 | 6 | 1 | **710** |
| 60 | 51 | BSA, candidemia, LA, AS | Blood | 09-07-16 | Kw74-16 | 1 | 7 | 4 | 17 | 22 | 4 | **696** |
| 61 | 52 | BSA, candidemia, AS | Blood | 10-07-16 | Kw75-16 | 1 | 7 | 4 | 17 | 11 | 3 | **695** |
| 62 | 53 | DM, BSA | Urine | 12-07-16 | Kw77-16 | 1 | 69 | 3 | 77 | 135 | 1 | **709** |
| 63 | 54 | UC, DM | Urine | 23-07-16 | Kw81-16 | 1 | 67 | 3 | 19 | 2 | 3 | **698** |

^a^Serial number of patients yielding multiple isolates are shown in Bold

^b^UC, urinary catheter; UTI, urinary tract infection; KD, kidney disease, US, Ureteric stent; DM, diabetes mellitus; BSA, broad spectrum antibiotic treatment; BSI, bloodstream infection; GI bleeding, gastro-intestinal bleeding; PEG, percutaneous endoscopic gastrostomy; PH, prostate hyperplasia; OC, ovarian cancer; AD, abdominal drain; LA, lung abscess; AS, abdominal surgery

^c^ET aspirate, endotracheal aspirate; PEG, percutaneous endoscopic gastrostomy; ABD fluid, abdominal drain fluid

^d^MLST-based DST, multi locus sequence type-based diploid sequence type; new DSTs detected in this study are shown in bold and DSTs shared between two isolates obtained from same or different patients are underlined.
