# Supplementary material for: Population structure and molecular genetic characterization of clinical Candida tropicalis isolates from a tertiary-care hospital in Kuwait reveal infections with unique strains
Source: PLoS One. 2017 Aug 30;12(8):e0182292. doi: 10.1371/journal.pone.0182292 (PMC5576731; doi:10.1371/journal.pone.0182292)
Supplement: S2 Table — (DOCX) [file pone.0182292.s002.docx]

**S2 Table**. **Salient features of various oligonucleotide primers used during MLST of *C. tropicalis* isolates in this study.**

| Primer | Target | Direction | Oligonucleotide sequence | Purpose | Product |
| --- | --- | --- | --- | --- | --- |
|  | gene |  |  |  | size (bp) |
| CTICLF | *ICL1* | Forward | 5’-ACTTTATCATTGGTGCCACCA-3’ | PCR |  |
| CTICLR | *ICL1* | Reverse | 5’-TCTGGTTGTTGGACGGTTTGA-3’ | PCR | 697 |
| CTICLFS | *ICL1* | Forward | 5’-AACCCAGAATCCGGCGACTT-3’ | Sequencing | N. A. |
| CTICLRS | *ICL1* | Reverse | 5’-GACGGTTTGACCGTATGCTCT-3’ | Sequencing | N. A. |
| CTMDRF | *MDR1* | Forward | 5'-TTGGCATTCACCCTTCCTGA-3' | PCR |  |
| CTMDRR | *MDR1* | Reverse | 5'- GGAGCACCAAACAATGGGAA -3' | PCR | 680 |
| CTMDRFS | *MDR1* | Forward | 5'-GCATTCACCCTTCCTGAATCT-3' | Sequencing | N. A. |
| CTMDRRS | *MDR1* | Reverse | 5'-CCAAACAATGGGAACACAGA-3' | Sequencing | N. A. |
| CTSAP2F | *SAPT2* | Forward | 5'-TTGAAGGACTTGCTATTTCCAA-3' | PCR |  |
| CTSAP2R | *SAPT2* | Reverse | 5'-ATAGTACCACTGGTAGCTGAA-3' | PCR | 691 |
| CTSAP2FS | *SAPT2* | Forward | 5'-GACTTGCTATTTCCAAGCTCA-3' | Sequencing | N. A. |
| CTSAP2RS | *SAPT2* | Reverse | 5'-CACTGGTAGCTGAAGGAGCA-3' | Sequencing | N. A. |
| CTSAP4F | *SAPT4* | Forward | 5’-TTCCTCATCTAGTCGTTGACA-3’ | PCR |  |
| CTSAP4R | *SAPT4* | Reverse | 5’-CAAAGTGATGGGAACATTATCA-3’ | PCR | 611 |
| CTSAP4FS | *SAPT4* | Forward | 5’-TCATCTAGTCGTTGACATGTT-3’ | Sequencing | N. A. |
| CTSAP4RS | *SAPT4* | Reverse | 5’-GATGGGAACATTATCATAAGTA-3’ | Sequencing | N. A. |
| CTXYRF | *XYR1* | Forward | 5’-TCTACTACTCCTACTATTCCTA-3’ | PCR |  |
| CTXYRR | *XYR1* | Reverse | 5’-GTTTGATAGTAGCACCTCTGA-3’ | PCR | 561 |
| CTXYRFS | *XYR1* | Forward | 5’-TACTCCTACTATTCCTACTATTA-3’ | Sequencing | N. A. |
| CTXYRRS | *XYR1* | Reverse | 5’-GATAGTAGCACCTCTGATCAA-3’ | Sequencing | N. A. |
| CTZWF | *ZWF1a* | Forward, | 5'-TGTCTTATGATTCCTTTGGTGA-3' | PCR |  |
| CTZWR | *ZWF1a* | Reverse, | 5'-TACCAATGTTATCGAAATAACCA-3' | PCR | 699 |
| CTZWFS | *ZWF1a* | Forward, | 5'-TATGATTCCTTTGGTGACCGT-3' | Sequencing | N. A. |
| CTZWRS | *ZWF1a* | Reverse, | 5'-GTTATCGAAATAACCACCTCTA-3' | Sequencing | N. A. |

N. A., not applicable
