# Supplementary material for: Population structure and molecular genetic characterization of clinical Candida tropicalis isolates from a tertiary-care hospital in Kuwait reveal infections with unique strains
Source: PLoS One. 2017 Aug 30;12(8):e0182292. doi: 10.1371/journal.pone.0182292 (PMC5576731; doi:10.1371/journal.pone.0182292)
Supplement: S3 Table — (DOCX) [file pone.0182292.s003.docx]

**S3 Table. Antifungal drug susceptibility testing data and the minimum inhibitory concentration (MIC) range, MIC_50_ and MIC_90_ values for 63 *C. tropicalis* isolates.**

| **Antifungal** | **No. of isolates detected as** | | | **MIC** | **MIC_50_** | **MIC_90_** |
| --- | --- | --- | --- | --- | --- | --- |
| **drug** | **Susceptible** | **Susceptible dose-dependent** | **Resistant** | **range (mg/L)** | **(mg/L)** | **(mg/L)** |
| Amphotericin B | 63 | 0 | 0 | 0.25 - 0.5 | 0.25 | 0.5 |
| 5-Flucytosine | 62 | 0 | 1 | 1.0 - 16 | 1 | 1 |
| Fluconazole | 63 | 0 | 0 | 1.0 - 1.0 | 1 | 1 |
| Voriconazole | 63 | 0 | 0 | 0.12-0.12 | 0.12 | 0.12 |
| Caspofungin | 63 | 0 | 0 | 0.25-0.25 | 0.25 | 0.25 |
| Micafungin | 63 | 0 | 0 | 0.06-0.06 | 0.06 | 0.06 |
