# Supplementary material for: Population structure and molecular genetic characterization of clinical Candida tropicalis isolates from a tertiary-care hospital in Kuwait reveal infections with unique strains
Source: PLoS One. 2017 Aug 30;12(8):e0182292. doi: 10.1371/journal.pone.0182292 (PMC5576731; doi:10.1371/journal.pone.0182292)

**Legend**

**S1 Fig. Agarose gel of PCR products using *C. tropicalis*-specific (CTROPF and CTROPR) primers and genomic DNA from reference strains of *C. albicans* (Lane CA), *C. dubliniensis* (Lane CD), *C. tropicalis* (Lane CT), *C. viswanathii* (Lane CV), *C. parapsilosis* (Lane CP), *C. orthopsilosis* (Lane CO), *L. elongisporus* (Lane LE), *C. glabrata* (Lane CG)*, C. nivariensis* (Lane CN) and *C. bracarensis* (Lane CB).** Water was used instead of DNA for a negative control (Lane BL) for PCR amplification reactions. Lane M is 100 bp DNA ladder and the positions of migration of 100 bp and 600 bp fragments are marked.


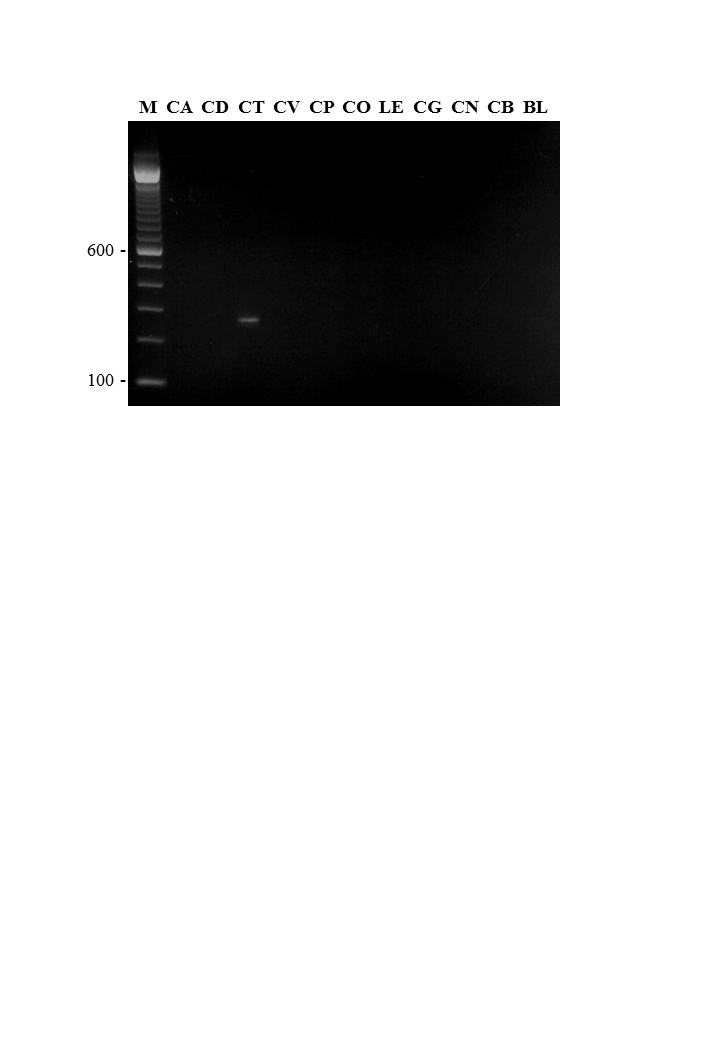

Supplement: S1 Fig — (DOCX) [file pone.0182292.s004.docx]
