# Supplementary material for: Population structure and molecular genetic characterization of clinical Candida tropicalis isolates from a tertiary-care hospital in Kuwait reveal infections with unique strains
Source: PLoS One. 2017 Aug 30;12(8):e0182292. doi: 10.1371/journal.pone.0182292 (PMC5576731; doi:10.1371/journal.pone.0182292)

**Legend**

**S2 Fig. An UPGMA-derived dendrogram with Tamura-Nei parameters based on ITS regions of rDNA sequence data from five *C. tropicalis* isolates from Kuwait together with reference *C. tropicalis* strain ATCC 750.** The numbers on the node branches are percent bootstrap frequencies from 1000 replicates.


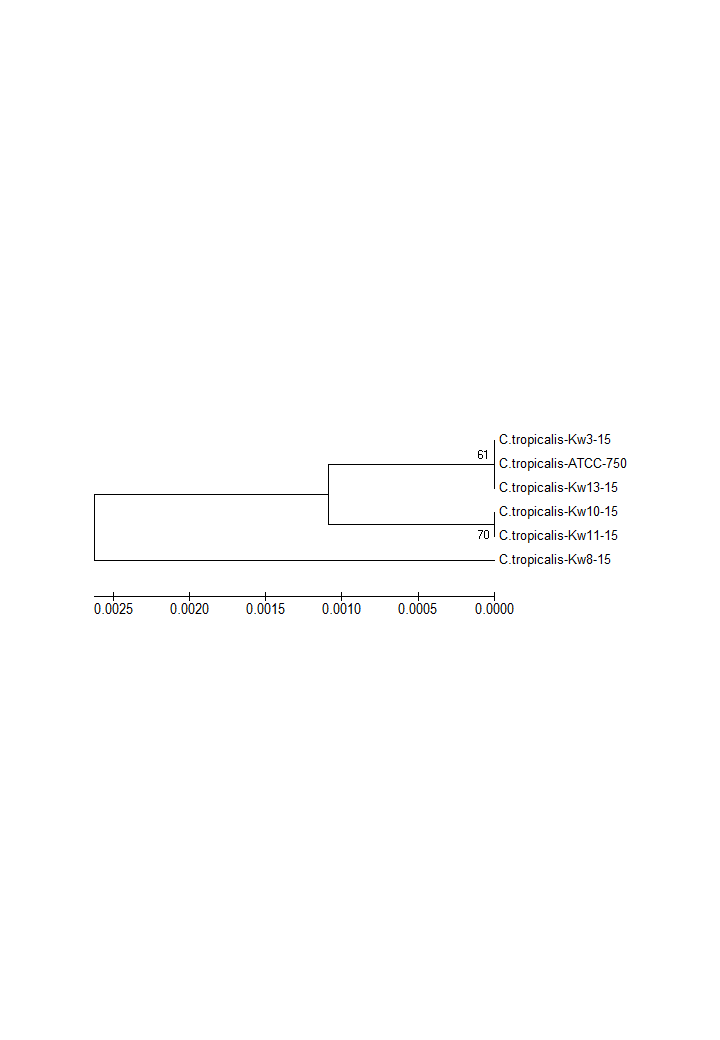

Supplement: S2 Fig — (DOCX) [file pone.0182292.s005.docx]
